# Video and phone visit use differed by language preference among U.S. Latino and Chinese adults during the first 9 months of the COVID-19 pandemic: a cross-sectional electronic health record study

**DOI:** 10.1186/s12913-024-11356-7

**Published:** 2024-08-07

**Authors:** Nancy P. Gordon, Teresa Y. Lin, Antonia Torreblanca, Mary E. Reed

**Affiliations:** 1 Division of Research, Kaiser Permanente Northern California, 4480 Hacienda Drive, Pleasanton, Oakland, CA 94588 USA; 2 The Permanente Medical Group, Oakland, CA USA; 3 Department of Adult and Family Medicine, Kaiser Permanente Oakland Medical Center, Oakland, CA USA

**Keywords:** Telehealth, Video visits, Limited English proficient, Visit preferences, Virtual care, Chinese adults, Latino adults, COVID-19

## Abstract

**Background:**

During the first nine months of the COVID-19 emergency, patients were encouraged to use virtual versus clinic visits if in-person care was not deemed necessary by clinical staff. This study examined the association of spoken language preference and ethnicity with use of video versus phone virtual visits by US Latino and Chinese adult patients who got care in the same healthcare system.

**Methods:**

We analyzed electronic health record data for four groups of adults aged 26-85y who had ≥ 1 primary or specialty care outpatient clinician visits during April-December 2020: 80,869 Latino adults preferring Spanish (LEP Latino); 214,765 Latino adults preferring English (non-LEP Latino); 23,430 Chinese adults preferring a Chinese dialect (LEP Chinese); and 49,710 Chinese adults preferring English (non-LEP Chinese). Prevalence of the following utilization outcomes were compared by language preference (LEP/non-LEP) within ethnicity and by ethnicity within language preference for four age subgroups (26-39y, 40-64y, 65-75y, and 76-85y): ≥ 1 virtual (video or phone) visit, ≥ 1 video visit, ≥ 1 phone visit, ≥ 1 clinic visit, video visits only, and phone visits only. We also compared ethnicity x language group differences within age subgroups using absolute difference and adjusted prevalence ratios derived from modified log-Poisson regression models that controlled for age and sex.

**Results:**

Among virtual users, LEP Latino and Chinese adults were significantly less likely to use video visits and more likely to use phone visits than non-LEP Latino and Chinese adults in the same age strata. The LEP/non-LEP difference in video visit use was significantly larger among Latino than Chinese patients, with no similar ethnic group difference observed for phone visits. Within the LEP and non-LEP language groups, Chinese adults were significantly more likely than Latino adults to use video visits and less likely to use phone visits.

**Conclusions:**

During the first nine months of the COVID-19 pandemic, uptake of video and phone virtual visits by Latino and Chinese adults significantly differed by LEP/non-LEP status within ethnicity and by ethnicity within LEP/non-LEP language group. These findings underscore the importance of disaggregating data by ethnicity and language preference when attempting to understand and study patient use of different virtual visit modalities.

**Supplementary Information:**

The online version contains supplementary material available at 10.1186/s12913-024-11356-7.

## Background

As part of the national COVID-19 emergency declared in March 2020, healthcare providers were encouraged to shift delivery of healthcare from in-person clinic visits to virtual (video or phone) visits when in-person care was not necessary [1]. This change was needed to reduce unnecessary staff and patient exposure to persons with the COVID-19 virus both in and during transit to the clinic setting at a time when protective masks were in very short supply for clinical staff and the public and to ensure that patients who required medical care did not forego it due to concern about becoming infected or difficulty coming into a medical facility. The United States (US) Center for Medicare & Medicaid Services (CMS) issued a waiver that enabled reimbursement for audio only and video virtual visits at the same rate as in-clinic visits, and many health insurers temporarily waived copays for virtual visits for their commercially insured members [2, 3]. Not surprisingly, dramatic increases in virtual (telephone and video) visits were observed across various settings during the first several months of the COVID-19 pandemic compared to earlier time periods [1, 4–6].

The shift to virtual visits, especially those conducted by video, raises important equity concerns with regard to healthcare access. Video visits have some advantages over telephone visits in that they can increase patients’ understanding, satisfaction, and feelings of personal connection with their clinicians [7, 8] and can improve clinicians’ diagnostic accuracy and ability to pick up nonverbal cues during the virtual encounter [9, 10]. However, video visits are more complicated for patients to engage in than phone visits, requiring following instructions to set up for the video visit as well as access to appropriate devices and high-speed internet [11, 12]. Lower rates of use of digital devices (e.g., computers, smartphones) and digital information technologies (e.g., internet, email, apps) and access to high-speed internet among older (≥ 65y) versus younger and middle-aged adults, Black and Latino versus White adults, and those of lower versus higher socioeconomic status have been well documented [13–16]. A survey of English-speaking adults found that Asian American adults have higher rates of digital device and digital information technology use and home broadband internet access than White adults [17]. In a survey of older adult health plan members, Chinese adults were more likely than other non-White groups to use patient portals [18]. More recent surveys of virtual visit users conducted during the COVID-19 pandemic found that among virtual (video or phone) visit users, Black, Latino, and Asian adults were less likely to choose video over phone visits [11, 19–21].

Evidence about the impact of having limited English proficiency (LEP) on use of digital information technologies is sparse, especially examination of the differences between LEP and non-LEP status within specific ethnic groups in the US. Using pooled data from the 2015–2018 California Health Interview Survey, Rodriguez et al*.* found that adults with LEP (defined as not able to speak English well) were less likely to have used the internet in the past 12 months [11]. Eberly et al*.* analyzed electronic health record (EHR) visit data for one health system during the early months of the pandemic and found that patients with LEP (defined as having a non-English preferred spoken language) were less likely than those without LEP to use virtual (versus in-person) visits, but that LEP did not significantly impact choice of video over phone visits [19]. Hsueh et al*.* found that among adult patients in Kaiser Permanente Northern California who had virtual visits during the first seven months of the pandemic, patients with LEP (defined as needing an interpreter] chose to have video (versus phone) visits less often than patients who did not need an interpreter, even after adjusting for age, race/ethnicity, and technology factors. However, this difference in video visit use was only significant among those who had no previous experience with video visits [22].

While these studies suggest that as a demographic subgroup, adult patients with LEP may be less likely to uptake video-based virtual visits, they are based on aggregated data that adjusts for, but does not stratify by, race/ethnicity and age group. Because of this, differences between LEP and non-LEP adult patients in use of patient portal features and video visits observed in previous studies may in part be associated with cultural and age-related preferences and digital information technology use. Spanish and Chinese are the two most commonly spoken non-English languages in the U.S. and California [23, 24]. The current study was designed to fill a gap in knowledge about the extent to which LEP status influenced choice of video versus phone virtual visits by US Latino and Chinese adult patients who received care from the same integrated healthcare delivery system during the first nine months following the declaration of the COVID-19 pandemic in 2020, and if differences by language status were seen, whether the magnitude of the differences varied by ethnicity.

## Methods

### Study design

This cross-sectional observational study used electronic health record (EHR) to describe differences in use of synchronous (“real time”) virtual visits conducted by video or phone by Latino and Chinese patients with and without LEP, restricted to a cohort of adult health plan members who had at least one coded virtual visit during April-December 2020. We abstracted ambulatory visits coded in the EHR as in-clinic, video, or phone appointments during this interval by departments known to have offered both video and phone visits during that period for all patients in the study cohort. Because of previously documented age group differences in readiness to use telemedicine [15], we examined whether language status (LEP vs. non-LEP) x ethnic group (Latino vs. Chinese) were associated with differences in video and phone visit use in four age strata (younger adults, middle-aged adults, younger seniors, and older seniors). This study was approved by the Kaiser Permanente Northern California (KPNC) Institutional Review Board, which waived the need to obtain Privacy Rule Authorization for use and disclosure of protected health information as allowed under {45 CFR 164.512(i)(1)(i)}.

### Setting

KPNC is an integrated healthcare delivery system that provides primary and specialty health care, laboratory tests, and pharmacy services to a racial/ethnically and sociodemographically diverse membership that includes over 3.2 million adults who mostly reside in the Greater San Francisco Bay Area, Sacramento area, Silicon Valley, and Central Valley. The KPNC adult membership has been shown to be very similar to the insured population of Northern California adults with regard to sociodemographic and health characteristics [25]. Scheduled telephone appointments have been available to members starting in 2008, and scheduled video visit appointments with primary care providers were offered starting in late 2014 [26], and by the April-December 2020 study period, were also expanded to include specialty care providers.

As a result of the COVID-19 emergency, starting in mid-March 2020 all members were encouraged to have video or phone visits rather than clinic visits when an in-person visit was not deemed necessary by their healthcare team. Both types of virtual visits were available with no visit co-pay, but video visits were encouraged as a way to provide a more personal connection with clinicians. Video visits required an internet-enabled device (computer, smartphone, or tablet) with a front-facing camera and microphone. Scheduled video visits could be joined by clicking on an emailed link to the patient portal website or apps for mobile devices. Members were able to sign in using their patient portal account username and password, or if they did not have an activated patient portal account or could not remember their sign-in credentials, they could gain temporary access by entering other identifying information. Detailed instructions for how to prepare for video visits, including a short video demonstration, were available on the health plan website and embedded in or linked to from the visit confirmation emails. Medical assistants and other frontline staff also conducted pre-video visit outreach (“virtual rooming”) to confirm that the patient still wanted a video visit and had the digital tools and knowledge needed to engage in a video visit and to ask whether a medical interpreter was going to be needed. More information about the health plan’s video visit program have been described elsewhere [27]. Members were able to convert from a video to phone appointment prior to or during the visit.

### Study population

The study cohort included 80,869 Latino adults whose preferred spoken language was Spanish (LEP Latino), 214,765 Latino adults whose preferred spoken language was English (Non-LEP Latino), 23,430 Chinese adults whose preferred spoken language was a Chinese dialect (LEP Chinese), and 49,710 Chinese adults whose preferred spoken language was English (Non-LEP Chinese). All adults were aged 26–85 years (y) in December 2020 and had been KPNC members during all of 2019 and 2020. The Latino adults were primarily identified from self-reported race and/or ethnicity data found in the EHR or self-reported Hispanic/Latino race/ethnicity captured in patient satisfaction and research surveys, with a tiny percentage (< 1.5%) assigned based on Spanish language preference. The Chinese adults were also primarily identified from EHR race and/or ethnicity or survey data, but additionally included members of known Asian race (but unknown ethnicity) who had a Chinese dialect indicated as preferred spoken language, Chinese as preferred written language, or a Chinese surname from a vetted surname list [28]. (See Table in Supplementary material for age strata-specific percentages of Latino and Chinese adults identified by these different methods). Assignment to LEP and non-LEP language group was based on member’s preferred spoken language in the EHR. Table 1 provides the numbers of adults in the four age strata we examined for each ethnicity x language group (26-39y, younger adults; 40-64y, middle-aged adults; 65-75y, younger seniors; 76-85y, older seniors) and the percentages of adults with female sex.

**Table 1:** Numbers of adults in the Latino and Chinese adult language subgroups used for virtual visit analyses

**Age group **^**a**^	**Latino**				**Chinese**			
	**Spanish preference**		**English preference**		**Chinese preference**		**English preference**	
	**N**	**% Female**	**N**	**% Female**	**N**	**% Female**	**N**	**% Female**
Adults who had ≥ 1 outpatient visit during Q2-4 2020^b^								
26-85y	80,869	54.5%	214,765	59.4%	23,430	62.7%	49,710	59.2%
26-39y	11,844	51.8%	74,227	63.7%	2,073	75.9%	11,207	66.4%
40-64y	52,590	54.4%	101,495	57.2%	10,417	65.7%	22,297	59.8%
65-75y	11,299	55.1%	27,610	56.7%	7,225	57.5%	11,850	54.3%
76-85y	5,136	59.6%	11,433	57.4%	3,715	57.2%	4,356	51.1%
Adults who had ≥ 1 virtual visit during Q2-4 2020^c^								
26-85y	75,671	54.6%	198,824	59.4%	21,238	62.6%	43,106	59.1%
26-39y	11,035	51.2%	69,220	63.4%	1,893	75.6%	9,910	66.3%
40-64y	49,661	54.4%	94,633	57.1%	9,492	65.2%	19,205	59.4%
65-75y	10,297	56.1%	24,925	57.1%	6,511	57.7%	10,211	54.4%
76-85y	4,678	60.5%	10,046	58.7%	3,342	57.2%	3,780	52.1%

Language preference is based on preferred spoken language listed in the member’s electronic health record. People assigned as Spanish or Chinese preference did not necessarily have an indicator that an interpreter was required for encounters. The majority of adults with Chinese preference spoke Mandarin or Cantonese dialects. Virtual visits are medical appointments conducted in “real time” by video or phone

^a^Age on December 1, 2020

^b^Corresponds to subgroups analyzed to produce Fig. 2

^c^Corresponds to subgroups analyzed to produce Figs. 3 and 4

### Study variables

Virtual visits were defined as scheduled outpatient appointments with a clinician that were coded in the EHR as video or phone visits. Clinic visits were scheduled outpatient appointments coded as having been conducted at the medical facility. As the focus of our study was on outpatient visits during Q2-4 (April through December) 2020, we created variables representing ≥ 1 virtual (video or phone) visit, ≥ 1 video visit, ≥ 1 phone visit, and ≥ 1 clinic visit during this period. We created these variables for all members who had at least one of these types of visits and then created variables for ≥ 1 video visit, ≥ 1 phone visit, and ≥ 1 clinic visit, restricting to those adults who had at least one virtual visit during this period. For comparison purposes, we created a variable representing ≥ 1 virtual visit for adults who had at least one outpatient visit during Q2-4 2019. We also created a variable characterizing use of different outpatient visit types in the 2020 study period among those with ≥ 1 outpatient visit (virtual visit only, clinic visit only, or both visit types) and among those with ≥ 1 virtual visit (virtual visit only or both virtual and clinic visits).

### Statistical analysis

All statistical analyses were performed using SAS v9.4 (SAS Institute, Cary, NC 2014). First, we used Proc Freq to produce percentages of adults with each virtual visit outcome for the four ethnicity x language subgroups, overall and by sex (male, female) within four age groups (26-39y, 40-64y, 65-75y, and 76-85y). Within each age group, we used cross-tabular comparisons with chi-square tests to assess whether there were statistically significant differences between (1) LEP versus non-LEP language status within Latino and Chinese ethnic groups, and (2) Latino versus Chinese ethnicity within LEP and non-LEP language status. We then calculated absolute differences (e.g., percentage in LEP Latino group minus percentage in non-LEP Latino group) who had these visit types. Because the ethnicity x language subgroups were so large, we set the criteria for meaningfully significant difference to be an absolute difference of ≥ 5 percentage points and chi-square *p*-value of < 0.001. Because there were minimal sex differences in percentages of virtual visit users who had ≥ 1 video visit and ≥ 1 phone visit, percentages shown in the Fig. 2 and Fig. 3 bar graphs, Table 2 absolute differences, and chi-square tests for differences were based on data for females and males combined.

We next compared the four ethnicity x language subgroups within each of the four age groups using adjusted prevalence ratios with 95% CIs derived from modified log-Poisson regression models that controlled for age as a continuous variable and sex. Adjusted prevalence ratios (aPRs) compare the prevalence of an outcome (e.g., visit type) in one group to that in another group (e.g., prevalence or percentage with ≥ 1 video visit in the LEP Latino group compared to that in the non-LEP Latino group) after adjusting for covariates. When the outcome being modeled is not a rare event (< 10% of the population), the aPR is a more appropriate measure of association than adjusted odds ratio (aOR) derived from a logistic regression model because the latter can inflate the strength of association [29].

## Results

**Fig. 1:**
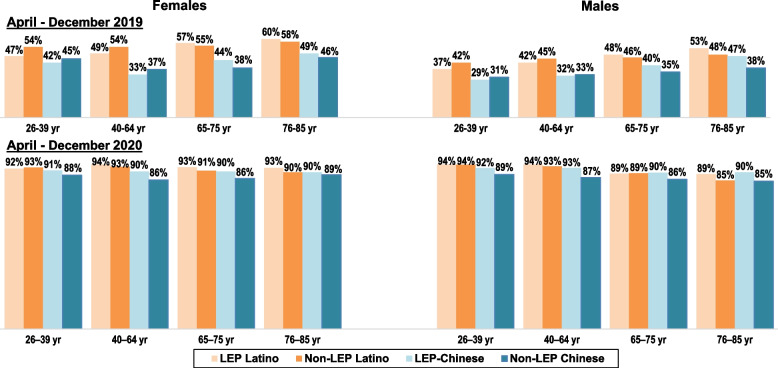
Latino and Chinese adults with ≥ 1 virtual visit, by preferred spoken language. Legend: Virtual visits are outpatient visits conducted by video or phone. LEP Latino adults have Spanish spoken language preference. LEP Chinese adults have a Chinese dialect spoken language preference. Non-LEP adults have English spoken language preference. All adults were health plan members during all of 2019 and 2020. Age group reflects age in December 2020. Comparisons show percentages of adults of Latino or Chinese ethnicity who were LEP or non-LEP who had ≥ 1 virtual visit during April – December 2019 and who had ≥ 1 virtual visit during April – December 2020, restricted to adults who had ≥ 1 outpatient visit during these time periods. Percentages are shown for four age groups and by sex. Subgroup denominators differ for 2019 and 2020 as they are based on adults who had ≥ 1 outpatient visit during that interval

As seen in Fig. 1, due to the COVID-19 emergency that resulted in outpatient care shifting from clinic-based visits to virtual visits unless an in-person visit was deemed necessary by healthcare providers, there was a dramatic increase in the percentages of adults with at least one outpatient visit who had virtual outpatient visits during April – December 2020 compared to the same interval in 2019. During the 2019 study period, 29% to 60% of adults with at least one outpatient visit had a virtual visit, with significant variation by age group, sex, and ethnicity. Specifically, among both LEP and non-LEP language groups, Chinese males and females were less likely than Latino males and females to have had a virtual visit, and in all four ethnicity x language groups, adults < 65y were less likely than those ≥ 65y to have had a virtual visit. In contrast, during the 2020 study period, most adults (range: 85% to 94%) who had at least one outpatient visit had at least one of those visits virtually, with little variation by LEP/non-LEP status, ethnicity, age group, and sex. Not shown is that nearly all virtual visits in 2019 were conducted by phone. Among adults who had ≥ 1 outpatient visit during that 2019 period, < 1% of those aged 65–85, ≤ 2% of those aged 40–64, and ≤ 3% of those aged 26–39 had a video visit, with no significant differences by sex or ethnicity x language group within these age groups.

**Fig. 2:**
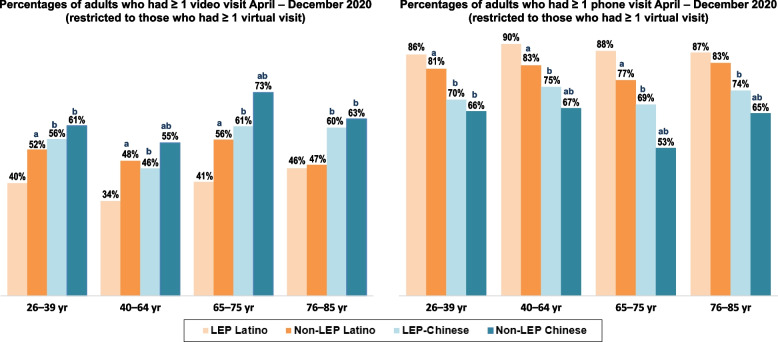
Latino and Chinese adults with ≥ 1 video and phone visit, by preferred spoken language. Legend: Virtual visits are outpatient visits conducted by video or phone. LEP Latino adults have Spanish spoken language preference. LEP Chinese adults have a Chinese dialect spoken language preference. Non-LEP adults have English spoken language preference. All adults were health plan members during all of 2019 and 2020. Age group reflects age in December 2020. Figure shows percentages of adults of Latino or Chinese ethnicity who were LEP or non-LEP who had ≥ 1 video visit ≥ 1 phone visit during April – December 2020, restricted to adults who had ≥ 1 virtual visit during this time period. Percentages are shown for four age groups

**Fig. 3:**
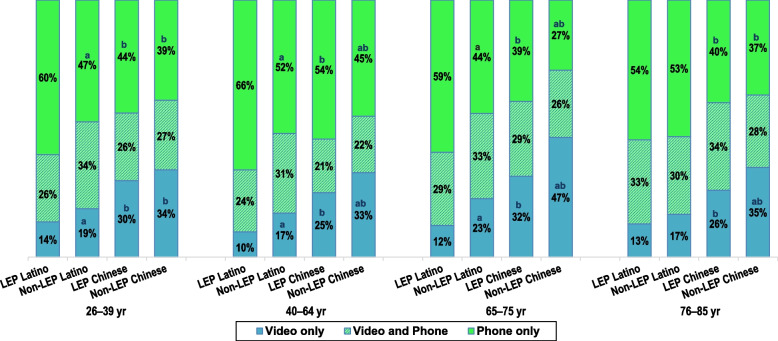
Latino and Chinese adults with video, phone, or both visit types, by preferred spoken language. : Virtual visits are outpatient visits conducted by video or phone. LEP Latino adults have Spanish spoken language preference. LEP Chinese adults have a Chinese dialect spoken language preference. Non-LEP adults have English spoken language preference. All adults were health plan members during all of 2019 and 2020. Age group reflects age in December 2020. Figure shows percentages of adults of Latino or Chinese ethnicity who were LEP or non-LEP who had only video visits, only phone visits, or both types of virtual visits during April – December 2020, restricted to adults who had ≥ 1 virtual visit during this time period. Percentages are shown for four age groups. ^**a**^ Within this age stratum and ethnic group, there was a meaningfully significant difference between LEP and Non-LEP adults based on a ≥ 5 percentage point difference between these subgroups and chi-square *p*-value < .001. ^**b**^ Within this age stratum and language status (LEP or non-LEP) group, there was a meaningfully significant difference between Chinese and Latino adults based on a ≥ 5 percentage point difference between these subgroups and chi-square *p*-value < .001

Figure 2 shows that among virtual users in 2020, percentages of adults who had ≥ 1 video visit and ≥ 1 phone visit meaningfully differed by LEP/non-LEP status in nearly all age groups. Figure 3 shows the same pattern for adults whose only virtual visits were by video or by phone. While 20–34% of these adults had both types of virtual visits, across all age groups, LEP and non-LEP Latino adults were less likely to have had ≥ 1 video visit and to have only had video visits than LEP and non-LEP Chinese adults, respectively. The converse was true for ≥ 1 phone visit, with LEP and non-LEP Latino adults more likely to have had ≥ 1 phone visit and to have only had phone visits than similarly aged LEP and non-LEP Chinese adults, respectively. In all age groups, LEP Latino and LEP Chinese adults were less likely than their non-LEP counterparts to have had ≥ 1 video visit and to have only had video visits and more likely to have had ≥ 1 phone visit and to have only had phone visits.

Table 2 summarizes the absolute differences between percentages by language status across age subgroups within the two ethnic groups and between Latino and Chinese adults within LEP and non-LEP status shown in Figs. 2 and 3. The aPRs in Table 2 show how prevalence of ≥ 1 video visit, ≥ 1 phone visit, and only having video and phone visits differed within age subgroups by LEP vs. non-LEP language status within ethnic group and by ethnic group within LEP and non-LEP language status after adjusting for age and sex within age subgroup. The aPRs confirm that after adjusting for age and sex, higher percentages of non-LEP than LEP adults in both ethnic groups had video visits and lower percentages of non-LEP than LEP adults had phone visits. For ≥ 1 video visit and only having video-based virtual visits, the non-overlapping 95% CIs for the aPRs also indicate that there were greater differences between LEP and non-LEP groups among Latino adults than among Chinese adults in all but the oldest age group. However, within age subgroups, the aPRs describing the difference in prevalence of ≥ 1 video visit by language status within these ethnic groups are smaller than for video visits and have similar 95% CIs.

**Table 2:** Comparisons of percentages of adults with ≥ 1 virtual visit in Q2-4 2020 who had these visits by video or phone, by preferred spoken language, within and across ethnic groups

Visit type	Age group	Non-LEP Latino vs LEP Latino		Non-LEP Chinese vs. LEP Chinese		LEP Chinese vs. LEP Latino		Non-LEP Chinese vs. Non-LEP Latino	
		**Absolute Difference**	**aPR (95% CL)**	**Absolute Difference**	**aPR (95% CL)**	**Absolute Difference**	**aPR (95% CL)**	**Absolute Difference**	**aPR (95% CL)**
≥ 1 video visit	26-39y	+ 12%	1.27 (1.24–1.30)	+ 5%	1.10 (1.05–1.14)	+ 16%	1.31 (1.25–1.37)	+ 9%	1.16 (1.14–1.18)
	40-64y	+ 14%	1.41 (1.39–1.43)	+ 9%	1.18 (1.15–1.22)	+ 12%	1.37 (1.33–1.40)	+ 7%	1.14 (1.12–1.16)
	65-75y	+ 15%	1.37 (1.33–1.40)	+ 12%	1.20 (1.17–1.23)	+ 20%	1.48 (1.44–1.53)	+ 17%	1.30 (1.28–1.33)
	76-85y	+ 1%	1.03 (0.99–1.07)	+ 3%	1.05 (1.01–1.09)	+ 15%	1.31 (1.26–1.37)	+ 17%	1.35 (1.31–1.39)
≥ 1 phone visit	26-39y	- 5%	0.93 (0.92–0.94)	- 4%	0.95 (0.92–0.98)	-16%	0.81 (0.78–0.83)	-15%	0.81 (0.80–0.82)
	40-64y	- 7%	0.92 (0.91–0.92)	- 8%	0.91 (0.90–0.93)	-15%	0.82 (0.81–0.83)	-16%	0.81 (0.80–0.82)
	65-75y	-11%	0.88 (0.87–0.89)	-15%	0.78 (0.76–0.80)	-19%	0.78 (0.77–0.80)	-23%	0.69 (0.68–0.71)
	76-85y	- 4%	0.96 (0.95–0.97)	- 9%	0.89 (0.87–0.92)	-13%	0.84 (0.82–0.86)	-18%	0.79 (0.77–0.81)
Video visits only	26-39y	+ 5%	1.43 (1.36–1.50)	+ 4%	1.11 (1.03–1.20)	+ 16%	2.29 (2.10–2.50)	+ 15%	1.82 (1.76–1.88)
	40-64y	+ 8%	1.78 (1.73–1.83)	+ 8%	1.25 (1.20–1.31)	+ 15%	2.76 (2.64–2.89)	+ 15%	1.90 (1.85–1.94)
	65-75y	+ 11%	1.85 (1.75–1.96)	+ 15%	1.47 (1.41–1.54)	+ 20%	2.56 (2.40–2.73)	+ 24%	2.04 (1.98–2.10)
	76-85y	+ 4%	1.28 (1.18–1.40)	+ 8%	1.29 (1.20–1.39)	+ 14%	2.06 (1.87–2.26)	+ 18%	2.07 (1.95–2.21)
Phone visits only	26-39y	-12%	0.82 (0.80–0.83)	- 5%	0.88 (0.3–0.93)	-16%	0.77 (0.73–0.81)	- 9%	0.82 (0.80–0.85)
	40-64y	-14%	0.79 (0.78–0.80)	- 9%	0.84 (0.82–0.86)	-12%	0.82 (0.80–0.83)	- 7%	0.87 (0.86–0.88)
	65-75y	-15%	0.75 (0.73–0.76)	-12%	0.69 (0.66–0.72)	-20%	0.67 (0.65–0.69)	-17%	0.62 (0.59–0.64)
	76-85y	- 1%	0.98 (0.95–1.01)	- 3%	0.92 (0.87–0.98)	-15%	0.73 (0.70–0.77)	-17%	0.69 (0.66–0.72)

**Fig. 4:**
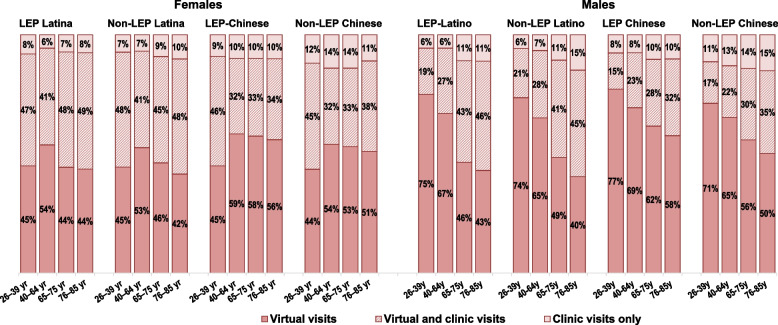
Latino and Chinese adults with virtual, in-person, or both visit types, by preferred spoken language. Legend: Virtual visits are outpatient visits conducted by video or phone. All adults were health plan members during all of 2019 and 2020. LEP Latino adults have Spanish spoken language preference. LEP Chinese adults have a Chinese dialect spoken language preference. Non-LEP adults have English spoken language preference. All adults were health plan members during all of 2019 and 2020. Age group reflects age in December 2020. Figure shows percentages of adults of Latino or Chinese ethnicity who were LEP or non-LEP who had only virtual visits, only clinic (in-person) visits, or both virtual and clinic visits during April – December 2020, restricted to adults who had ≥ 1 outpatient visit during this time period. Percentages are shown for females and males in four age groups

All comparisons are restricted to adults who were health plan members January 2019 through December 2020 and had ≥ 1 virtual (video or phone) visit during Q2-4 2020. Age group reflects age in December 2020. Percentages of adults in these subgroups with these visit types are found in Figs. 3 and 4. Non-LEP Latino: Latino adults with English indicated in electronic health record (EHR) as their preferred spoken language. LEP Latino: Latino adults with Spanish indicated as their preferred spoken language. Non-LEP Chinese: Chinese adults with English indicated as their preferred spoken language. LEP Chinese: Chinese adults with a Chinese dialect indicated as their preferred spoken language

Absolute difference was calculated by subtracting the percentage with this type of visit in the second named subgroup from the percentage with this type of visit in the first named subgroup. aPR: Adjusted prevalence ratio compares percentages with this type of visit in the first named subgroup to the percentages with this type of visit in the second named subgroup after adjusting for sex and age as a continuous variable using a modified log-Poisson regression model

Adjusted PRs comparing video visit use among Chinese vs. Latino adults within language groups confirm that after adjusting for age and sex, Chinese adults in both language groups were more likely to have had a video visit and less likely to have had a phone visit than similarly aged Latino adults. The aPRs and 95% CIs comparing any videos and only video visits by ethnicity within language group indicate a greater disparity between the Chinese and Latino adults with LEP group than between those with non-LEP. However, the aPRs showing differences by ethnic group in any phone visits and only phone visits are similar across LEP and non-LEP groups.

Finally, we examined the percentages of females and males in the LEP and non-LEP Latino and Chinese ethnicity x language groups who had only virtual visits during the April to December 2020 study period **(**Fig. 4**)**. Among both sexes, the percentages of virtual visit users with ≥ 1 clinic visit did not significantly differ by LEP/non-LEP status within each ethnic group based on the greater than ± 5 percentage point difference criteria. Approximately half of Latina females in the 26-39y, 65-74y, and 76-85y age subgroups and approximately 44% in the 40-64y age subgroup had ≥ 1 clinic visit. Among Chinese females, the percentage of those in the 26-39y age subgroup with ≥ 1 clinic visit was the same as for Latina females, but the percentages of middle-aged and older females were significantly lower than similarly aged Latina females (range: 35% to 43%). The percentages of Latino and Chinese males in the 26-39y and 40-64y age groups who had ≥ 1 clinic visit (range: 21% to 30%) were significantly lower than those of females in these age groups. Similar to the females, Chinese males in the 65-74y and 75-85y age groups were less likely than Latino males to have had ≥ 1 clinic visit in addition to virtual visits during this period.

Figure 4 also shows that young adult (26-39y) LEP and non-LEP Latina and Chinese females were significantly less likely than their middle-aged (40-64y) counterparts to have only virtual visits during the study period. In the older age groups, the percentages of LEP and non-LEP Latina females with only virtual visits were similar to those of Latinas in the younger adult group, while the percentages of LEP and non-LEP Chinese females with only virtual visits were similar to those of the middle-aged Chinese groups. A different pattern of only virtual visits is seen among males. LEP and non-LEP Latino and Chinese males in the young adult subgroup were more likely than their middle-aged counterparts to have had only virtual visits, who were more likely than their counterparts in the older age groups to have had only virtual visits. Comparing LEP and non-LEP Latino and Chinese males and females within age groups, younger and middle-aged males were more likely than similarly aged females to have had only virtual visits, while a sex difference was not seen in the older age groups.

## Discussion

Currently, there are few published EHR- and survey-based studies that have examined the association of having limited English proficiency (LEP) on virtual (video and phone) and video visit use during the early months of the COVID-19 pandemic in multiethnic, multilingual U.S. adult populations. Among these, two compared video visit use among adults with English versus non-English spoken language preference [30] or need for an interpreter [22] but did not use data disaggregated by preferred non-English language and ethnic group. Another study conducted by Hsueh et al. compared use of video visits by LEP (interpreter needed) adults with Spanish, Chinese, and other spoken language preferences, but only included LEP adults who had self-scheduled these visits through the health plan’s patient portal and also controlled for multiple sociodemographic and health-related characteristics in the comparisons [31].

In our EHR-based study, we used cohorts of LEP and non-LEP Latino and Chinese adults to examine the extent to which both LEP status and ethnicity influenced choice of video versus phone visits among virtual visit users who received care from the same integrated healthcare delivery system and had the same free (no co-pay) access, encouragement, and organizational support, including interpreter services, to use video visits. We found that the prevalence of virtual (video or phone) visit utilization among adult patients who had at least one outpatient visit during the first nine months of the COVID-19 pandemic, when patients were being encouraged to substitute virtual for in-person clinic visits, was similar across the four ethnicity x language groups.

Among virtual visit users aged 26-75y, Latino adults who preferred to speak Spanish and Chinese adults who preferred to speak a Chinese dialect were less likely to use video visits and more likely to use phone visits than Latino and Chinese adults in the same age groups whose preferred spoken language was English. Further, the difference between language groups in prevalence of video visit use was significantly larger among Latino than Chinese patients, while no similar ethnic group difference was observed for prevalence of phone visit use. We also showed that differences by language status were mediated by ethnicity. Specifically, within the LEP and non-LEP language groups, Chinese adults were significantly more likely than Latino adults to have used video visits and significantly less likely to have used phone visits. In the 76-85y age group, we observed the same differences in pattern of video and phone visit use between Latino and Chinese adults within LEP and non-LEP language groups but saw no significant difference by language status within ethnic group. Finally, we found that during the study period, substantial percentages of adults in all ethnicity x language groups had both types of virtual visits and had in-person clinic visits in addition to virtual visits, with younger and middle-aged adult males in all ethnicity x language groups most likely to have had only virtual visits, followed by middle-aged females.

Our study results have implications for service planning, reimbursement policy, and research. With regard to service planning, adult Latino patients may be better able to use or more comfortable using phone-based virtual visits than video visits, while adult Chinese patients may be better able to use or more comfortable using video visits than Latino patients. This suggests that to provide optimal patient-centered care, clinicians and healthcare providers should offer but not try to push all patients to use video visits. Further research is needed to increase understanding of factors that are driving ethnic and language group differences in video visit use in order to inform healthcare system interventions that could address potentially modifiable barriers to this mode of healthcare access.

Ethnic and language group differences in preference for phone-based virtual also have implications for reimbursement policy. If healthcare providers are reimbursed for video visits but not phone visits, this could adversely affect healthcare providers who provide care to large numbers of patients who prefer phone-based virtual care or lack access to the digital technologies and skills required for video visits. Differential reimbursement practices could also result in healthcare providers pushing patients to use video visits even when the patient does not prefer or feel comfortable using that modality of communication or finds it less convenient and potentially more expensive.

Finally, our study results have implications for how to conduct scientific and operations research to study differences in use of different types of virtual visits in multiethnic and multilingual adult populations. Specifically, to learn whether and how limited English proficiency affects use of and opinions about video and phone visits, it is important to collect and analyze data for patients with LEP disaggregated by ethnic group rather than solely examining for differences in use by language status using models that control for ethnicity, language status, and other demographic and healthcare coverage characteristics. Similarly, to learn whether use of and opinions about different types of virtual visits differ across racial and ethnic groups, it is important to collect and analyze data by ethnicity, preferred spoken language, and age group.

Our study has several strengths. First, our study subgroups were very large. This not only enabled us to produce virtual visit prevalence statistics with tight confidence intervals separately for LEP and non-LEP adults within the Latino and Chinese ethnic groups, but also to compare the four ethnicity x language groups within young, middle-aged, and older adult age strata. Second, all adults in our study received outpatient care through the same integrated healthcare delivery system, had similar access to video and phone visits, technical support and staff outreach prior to video visits, and interpreter services available for both types of virtual appointments. Third, the vast majority of virtual visits were offered without cost-sharing (co-pays) during the study period, removing potential financial barriers and incentives for choosing one modality over the other. Fourth, while video visits were conducted using a secure platform, members were able to sign into a video appointment without having a patient portal account or using their patient portal credentials. Finally, our visit statistics were based on outpatient visits coded by visit type (video, phone, or clinic) identified from electronic health records rather than self-reported survey data.

We also acknowledge that our study has some limitations. First, we assigned adults to LEP and non-LEP language groups based on preferred spoken language listed in the EHR. It is possible, that some adults whose EHR indicated a non-English spoken language preference could communicate somewhat well in English. We also are not fully confident that all of the adults we classified as non-LEP spoke and understood English very well. Anecdotally, we have heard from clinicians that some of their patients who do not speak English very well may prefer to attempt to communicate in English rather than to use a medical interpreter. Additionally, some Latino and Chinese adults listed as having English spoken language preference may have a primary care physician or clinician support staff who speaks enough of their language to carry on a conversation or are assisted in communicating with medical staff by a bilingual family member or friend. Second, while all Latino adults were assigned to that ethnicity based on self-reported data, some Chinese adults with known Asian race were assigned to Chinese ethnicity based on surname or language preference. Third, while we believe that during the study period, all members were offered the choice of a virtual visit by video or phone when an in-person visit was not deemed necessary, we cannot be certain that this occurred in all circumstances. We know that members who were self-scheduling visits through the patient portal saw the option for video or phone modality, and the protocol for all health plan Call Center agents was to offer members requesting an appointment a video visit first and then a phone visit if they did not want a video visit. Fourth, we only captured virtual visits during the first nine months of the COVID-19 pandemic. As the pandemic went on, virtual visits became more normative, clinicians and staff gained more experience and become more comfortable using video visits, and healthcare system workflows and patient supports for video visits improved across all medical facilities. It is possible that as a result of these changes, we may have seen smaller differences by language status and ethnicity if we had studied a longer time period. Fifth, we were unable to link data about whether the healthcare provider was able to conduct visits in the patient’s preferred language nor whether the patient required an interpreter for video and phone visits. Sixth, we lacked data to examine reasons for differences in video versus phone visit use by LEP/non-LEP status and ethnicity that could elucidate the extent to which the differences we found result from healthcare access barriers, such as being offered a video visit or difficulty using the video visit platform, or patient factors, including disparities in educational attainment, access to needed digital technologies, familiarity with video chat, and patient preferences. However, that was beyond the scope of this study. Finally, while we believe that conducting this study with a cohort of patients who all received care from the same Northern California healthcare system during the same time interval was a strength of this study, as previously described, this also potentially limits generalizability of the results to other healthcare settings, other patient populations, and future study periods when the universal waiver of co-pays for virtual visits during the early years of the COVID-19 pandemic may be lifted.

## Conclusions

During the first nine months of the COVID-19 pandemic when most adult outpatient visits were conducted virtually, there were significant differences in type of virtual visits used by Latino and Chinese adult virtual visit users with LEP (preferred spoken language was Spanish or a Chinese dialect, respectively) compared to those who were non-LEP (preferred spoken language of English). In both ethnic groups, virtual visit users aged 26-85y with LEP were less likely than those with non-LEP to have had video visits and more likely to have had phone visits. However, within LEP and non-LEP language groups, Chinese adults were more likely than Latino adults to have used video visits and less likely to have used phone visits. Results underscore the importance of disaggregating data by ethnicity and language preference when attempting to understand and study patient use of different virtual visit modalities. Further research is needed to understand and address factors driving lower utilization of video visits among Latino versus Chinese adults and among adults with limited English proficiency in both ethnic groups to promote health and healthcare equity in these vulnerable patient populations.

### Supplementary Information

Supplementary Material 1.

## Data Availability

The datasets analyzed during the current study are not publicly available due to the Kaiser Permanente Northern California (KPNC) Institutional Review Board not providing approval for variables derived from electronic health data to be placed in a public access repository. These data are available from the corresponding author (NPG) or the KPNC Division of Research Data Sharing Workgroup (DOR-DataSharingWorkgroup@kp.org).

## Abbreviations

aPR: Adjusted prevalence ratio
CI: Confidence interval
CMS: Center for Medicare & Medicaid Services
EHR: Electronic health record
KPNC: Kaiser Permanente Northern California
LEP: Limited English proficient
Q: Quarter
US: United States
y: Years
