# Supplementary material for: Video and phone visit use differed by language preference among U.S. Latino and Chinese adults during the first 9 months of the COVID-19 pandemic: a cross-sectional electronic health record study
Source: BMC Health Serv Res. 2024 Aug 7;24:900. doi: 10.1186/s12913-024-11356-7 (PMC11304802; doi:10.1186/s12913-024-11356-7)
Supplement: Supplementary file 1 — Supplementary Material 1. [file 12913_2024_11356_MOESM1_ESM.docx]

| **Table S1. Data sources used to identify Latino and Chinese study groups** | | | | | | | | |
| --- | --- | --- | --- | --- | --- | --- | --- | --- |
|  | **Limited English Proficient** | | | | **Non-Limited English Proficient** | | | |
|  | **EHR R/E** | **Questionnaire** | **EHR Language** | **Surname** | **EHR R/E** | **Questionnaire** | **EHR Language** | **Surname** |
| **Latino adults** |  |  |  |  |  |  |  |  |
| **Women** |  |  |  |  |  |  |  |  |
| 26-39 years, % | 98.6% | 0.5% | 0.8% | 0.0% | 97.1% | 2.9% | 0.0% | 0.0% |
| 40-64 years, % | 97.9% | 1.2% | 0.9% | 0.0% | 93.1% | 6.8% | 0.0% | 0.0% |
| 65-75 years, % | 98.1% | 1.5% | 0.4% | 0.0% | 87.6% | 12.4% | 0.0% | 0.0% |
| 76-85 years, % | 97.8% | 1.7% | 0.4% | 0.0% | 85.1% | 14.9% | 0.0% | 0.0% |
|  |  |  |  |  |  |  |  |  |
| **Men** |  |  |  |  |  |  |  |  |
| 26-39 years, % | 95.7% | 1.4% | 2.9% | 0.0% | 97.3% | 2.7% | 0.0% | 0.0% |
| 40-64 years, % | 97.1% | 1.2% | 1.7% | 0.0% | 96.1% | 3.9% | 0.0% | 0.0% |
| 65-75 years, % | 98.4% | 1.2% | 0.4% | 0.0% | 90.7% | 9.3% | 0.0% | 0.0% |
| 76-85 years, % | 98.6% | 1.3% | 0.1% | 0.0% | 87.8% | 12.2% | 0.0% | 0.0% |
|  |  |  |  |  |  |  |  |  |
| **Chinese adults** |  |  |  |  |  |  |  |  |
| **Women** |  |  |  |  |  |  |  |  |
| 26-39 years, % | 61.3% | 1.0% | 7.8% | 0.0% | 70.0% | 9.0% | 0.9% | 20.1% |
| 40-64 years, % | 62.5% | 5.4% | 2.0% | 0.0% | 67.6% | 17.7% | 0.8% | 13.8% |
| 65-75 years, % | 77.5% | 6.2% | 16.3% | 0.0% | 71.3% | 20.5% | 0.8% | 7.5% |
| 76-85 years, % | 84.8% | 6.7% | 9.2% | 0.0% | 71.2% | 24.2% | 0.5% | 4.1% |
|  |  |  |  |  |  |  |  |  |
| **Men** |  |  |  |  |  |  |  |  |
| 26-39 years, % | 35.9% | 2.6% | 61.5% | 0.0% | 50.3% | 10.7% | 0.9% | 38.1% |
| 40-64 years, % | 55.5% | 3.4% | 41.1% | 0.0% | 62.9% | 10.2% | 1.0% | 25.9% |
| 65-75 years, % | 75.4% | 5.4% | 19.2% | 0.0% | 73.8% | 15.3% | 0.9% | 10.0% |
| 76-85 years, % | 83.3% | 7.2% | 9.5% | 0.0% | 74.3% | 20.3% | 0.7% | 4.7% |
| EHR R/E: Race and ethnicity fields in electronic health record (EHR); Questionnaire: Surveys or clinical/administrative intake forms; EHR Language: Preferred spoken or written language fields in EHR; Surname: Assigned based on last name if identified as Asian race from EHR or questionnaire data but no specified ethnicity. Ethnicity assignment gave priority to EHR R/E, followed by questionnaire data, EHR language preference, and finally surname (Chinese only). | | | | | | | | |
